# Iterative reconstruction of CT images via Score Function

**Published:** 2023-10-31

**Authors:** Wenxiang Cong, Wenjun Xia, Ge Wang

**Affiliations:** Biomedical Imaging Center, Department of Biomedical Engineering, Rensselaer Polytechnic Institute, Troy, NY 12180

**Keywords:** Computed tomography (CT), radiation dose reduction, image reconstruction, maximum a posteriori (MAP) estimation, machine learning, score function

## Abstract

Computed tomography (CT) reconstructs sectional images from X-ray projections acquired from multiple angles around an object. In the case of low-dose or sparse-view scans, the image reconstruction using classical tomographic algorithms can produce severe noise and artifacts. To address this issue, we present a deep learning-based image reconstruction method derived from maximum a posteriori (MAP) estimation. In the Bayesian statistics framework, the gradient of logarithmic probability density distribution of the image, i.e., the score function, plays a crucial role, contributing to the process of image reconstruction. We develop an improved score matching (ISM) solution for the image reconstruction by leveraging Gaussian mixture to characterize noise distributions. The reconstruction algorithm with the score function theoretically guarantees the convergence of the iterative process. Our results also show that this reconstruction method can produce higher quality images compared to state-of-the-art reconstruction methods.

## 1. Introduction

X-ray computed tomography (CT) is a crucial imaging modality used in various fields, including medical imaging, security and industrial applications [1]. Filtered back-projection (FBP) is an analytical image reconstruction method for CT [2]. In the cases of low dose or sparse-view CT scans, FBP often generates noise and artifacts in reconstructed images [3–5]. To address this challenge, iterative methods were developed to incorporate prior knowledge of images and imaging system. These methods leverage a statistical distribution of photons to improve CT image quality [5, 6]. Iterative algorithms can also incorporate compressed sensing (CS) techniques to enforce the sparsity of reconstructed images for image denoising and deblurring. Total variation (TV) minimization is a typical regularization method for the image reconstruction [4, 6]. However, TV method often oversmoothes textured regions and eliminates some subtle details. The low-dimensional manifold model (LDMM) crops an image into patch set to form a point cloud sampled from a low-dimensional manifold embedded in a high-dimensional ambient space, providing a new regularization way by minimizing the dimensionality of the corresponding image patch manifold [7]. The patch manifold of images typically exhibits a low-dimensional structure and yet accommodates rich features [8]. Utilizing LDMM prior knowledge of images, iterative reconstruction methods would lead to improvements in image quality [7]. But, the LDMM requires high computational cost for image reconstruction.

Emerging deep learning is a powerful technique for various tasks through learning and inference in a data-driven fashion [9]. These learning techniques can perform various types of uncertainty estimation and data modeling [10]. In this paper, we develop a deep-learning-based image reconstruction method for CT image reconstruction. The X-ray projection data can be modeled as a Poisson distribution, which allows us to reconstruct the image using maximum a posteriori (MAP) estimation. In the Bayesian statistics framework, the gradient of logarithmic probability density function of the image, called the score function, plays a crucial role, contributing to the process of image reconstruction. We develop an improved score matching (ISM) solution for the image reconstruction by leveraging Gaussian mixture to characterize noise distributions. The score function can be modeled by data learning based on parameterized deep neural networks. The score function essentially preserves the same amount of information as the associated probability density function, representing a promising research avenue towards better data modeling, generation and inference, which differs significantly from traditional regularization functions assumed artificially, such as piecewise constants, image sparsity, and low dimensionality. Therefore, this data-driven method can obtain optimal prior knowledge of the image probability density distribution for CT image reconstruction.

The rest of this paper is organized as follows. Section II details the methodology, formulating and estimating the score function of an underlying image distribution, especially presenting a deep neural network to learn the score function from a training dataset. The image reconstruction is formulated using a statistical iterative process incorporated with the scoring function. The convergence of the iterative sequence is proven through rigorous mathematical analysis. In Section III, the proposed ISM-based reconstruction method is evaluated by comparing with state-of-the-art image reconstruction methods such as analytic filtered backprojection (FBP) reconstruction method, the simultaneous algebraic reconstruction technique (SART), advanced methods like SART with total variation (TV) and SART with low-dimensional manifold model (LDMM) regularization methods, as well as the popular score function-based image reconstruction methods. The representative image reconstruction results are reported on numerical experiments and clinical CT data, demonstrating the feasibility and effectiveness of our ISM-based image reconstruction method. Finally, in Section IV, relevant issues are discussed.

## 2. Methodology for CT image reconstruction

### 2.1. Maximum a posteriori (MAP) image reconstruction

In X-ray imaging, the number of X-ray photons recorded by a detector element is a random variable ξ, which can be modeled as the Poisson distribution [2]:

(1) $$p\left(\xi =y_{i}\right)=\frac{{\left({\bar{y}}_{i}\right)}^{y_{i}}}{y_{i}!}\text{exp}\left(-{\bar{y}}_{i}\right),$$

where ${\bar{y}}_{i}$ is the expectation value of recorded X-ray photons along a path l from the X-ray source to the $i^{\text{th}}$ detector element, and obeys the Beer-Lambert law:

(2) $${\bar{y}}_{i}=n_{i}\text{ exp}\left(-\int_{l}\mu (\vec{r})dl\right),$$

where $n_{i}$ is the number of X-ray photons recorded by the $i^{\text{th}}$ detector element in the blank scan (without any object in the beam path), and μ(r) is the linear attenuation coefficient distribution within an object to be reconstructed. For the numerical implementation, Eq. (2) is discretized as,

(3) $${\bar{y}}_{i}=n_{i}\text{ exp}\left(-A_{i}\mu \right),$$

where μ is a vector of pixel values in the linear attenuation coefficient image, and $A_{i}$ is weighting coefficients of the pixel values along the $i^{\text{th}}$ beam path. Assuming that measurements are independent, the likelihood function of the X-ray projection data can be obtained by

(4) $$p(Y|\mu )=\prod_{i=1}^{m}\frac{{\left({\bar{y}}_{i}\right)}^{y_{i}}}{y_{i}!}\text{exp}\left(-{\bar{y}}_{i}\right),$$

where $Y={\left(y_{1},y_{2},\cdots ,y_{m}\right)}^{T}$ is the number of photons measured by detectors, and m is the total number of X-ray detectors. Based on the Bayesian theorem: p(μ∣Y)p(Y)=p(Y∣μ)p(μ), the image reconstruction can be performed using the maximum a posteriori (MAP) estimation, which is equivalent to the following minimization problem:

(5) $$\mu_{\text{min}}=\underset{\mu }{\text{arg min}}\left(\sum_{i=1}^{m}\left[{\bar{y}}_{i}-y_{i}\text{ log}\left({\bar{y}}_{i}\right)\right]-\text{log }p(\mu )\right),$$

where log(p(μ)) is a logarithmic probability density of an attenuation image expressing the prior knowledge about images μ in a specific application domain. Combining Eqs. (3)–(5), we have

(6) $$\mu_{\text{min}}=\underset{\mu }{\text{arg min}}\left(\overset{m}{\underset{i=1}{\sum }}\left[n_{i}\text{ exp}\left(-A_{i}\mu \right)+y_{i}A_{i}\mu \right]-\text{log }p\left(\mu \right)\right).$$

Applying the second-order Taylor approximation, Eq. (6) can be simplified to a quadratic optimization problem [6]:

(7) $$\mu_{\text{min}}=\underset{\mu }{\text{arg min}}\left[\frac{1}{2}{\left(A\mu -b\right)}^{T}D\left(A\mu -b\right)-\text{log }p\left(\mu \right)\right]$$

where A is the m×n system matrix composed of the row vectors $A_{1},A_{2},\cdots ,A_{m}$, and D is the diagonal matrix of the form $\text{diag}\left(y_{1},y_{2},\cdots ,y_{m}\right)$. The optimization problem defined by Eq. (7) can be solved using the gradient-based iterative method in the algebraic reconstruction technique framework:

(8) $$\mu^{k+1}=\mu^{k}-\left(\omega A^{T}D\left(A\mu^{k}-b\right)-\sigma \nabla \text{log }p\left(\mu^{k}\right)\right),\;k=1,2,\cdots$$

where ∇log p(μ) is the score function of the probability density distribution with respect to the current image, and ω and σ are parameters for trade-offs between the data fidelity and sample plausibility.

### 2.2. Convergence of score-function-based image reconstruction

For the convergent analysis of the iteration scheme (8), we assume that A is a m×n system matrix and has rank n. The probability density function p(μ) is assumed to be sufficiently smooth, and the Hessian matrix $\nabla^{2}\text{log }p\left(\mu \right)$ of the logarithmic probability density function has bounded eigenvalues [11, 12], denoted by $\left|h_{i}\right|<C,\;i=1,2,\cdots ,n$. Based on these assumptions, we have the following Lemma for the convergence of the iteration scheme (8).

**Lemma 1:** The iteration scheme in Eq. (8) is convergent.

**Proof:** Based on the iteration procedure Eq. (8), we obtain

(9) $$\mu^{k+1}-\mu^{k}=\left(I-\omega A^{T}DA\right)\left(\mu^{k}-\mu^{k-1}\right)+\sigma \left[\nabla \text{log }p\left(\mu^{k}\right)-\nabla \text{log }p\left(\mu^{k-1}\right)\right],$$

From the mean value theorem of a multivariate function, there exists a vector ξ such that,

(10) $$\text{log }p\left(\mu^{k}\right)-\text{log }p\left(\mu^{k-1}\right)=\nabla \text{log }p\left(\xi \right)\left(\mu^{k}-\mu^{k-1}\right).$$

From Eqs. (9–10), we obtain

(11) $$\mu^{k+1}-\mu^{k}=\left(I-\omega A^{T}DA+\sigma \nabla^{2}\text{log }p\left(\xi \right)\right)\left(\mu^{k}-\mu^{k-1}\right),$$

where $\nabla^{2}\text{log }p\left(\xi \right)$ is the Hessian matrix of the logarithmic probability density function and is symmetric. Since ${\left(Ax\right)}^{T}DAx=\sum_{i=1}^{m}y_{i}{\left(A_{i}\cdot x\right)}^{2}>0$ for any nonzero vector $x\in R^{n}$, the matrix $A^{T}DA$ is positive definite, denoting its smallest and largest eigenvalues as $\lambda_{\text{min}}$ and $\lambda_{\text{max}}$ respectively. From Eq. (11), it is easy to find that by choosing the parameters ω and σ to satisfy $0<\sigma <\omega r_{\text{min}}/C$ and $\sigma C/r_{\text{min}}<\omega <\left(2-\sigma C\right)/r_{\text{max}}$, there exist a positive constant 0<q<1 such that $\left\|\mu^{k+1}-\mu^{k}\right\|\leq q\left\|\mu^{k}-\mu^{k-1}\right\|$, and $\left\|\mu^{k+1}-\mu^{k}\right\|\leq q^{k}\left\|\mu^{1}-\mu^{0}\right\|$. Hence, according to the Cauchy convergence criterion, the image sequence $\left\{\mu^{k}|k=0,1,\cdots \right\}$ must be convergent.

### 2.3. Score function estimation

The score function is the gradient of the logarithmic probability density with respect to images, i.e., $\nabla \text{log }p_{\text{data}}\left(x\right)$. The unknown score function in Eq. (8) must be estimated in advance to perform the image reconstruction. If a known dataset sampled from a data distribution $p_{\text{data}}\left(x\right)$ is available, the score function $\nabla \text{log }p_{\text{data}}\left(x\right)$ can be estimated using the score matching method, which is introduced by Hyvärinen (2005) [13]. The score matching method is to train a neural network model $s_{\theta }\left(x\right)$ via optimizing model parameters θ to best matches the score function $\nabla \text{log }p_{\text{data}}\left(x\right)$. The task can be performed by minimizing the following objective function:

(12) $$E_{p_{\text{data}}}\left[{\left\|s_{\theta }(x)-\nabla \text{log }p_{\text{data}}(x)\right\|}_{2}^{2}\right].$$

It has been demonstrated that above objective function is equivalent to the following [14]:

(13) $$E_{p(\tilde{x}|x)p_{\text{data}}(x)}\left[{\left\|s_{\theta }(\tilde{x})-\nabla_{\tilde{x}}\text{log }p(\tilde{x}|x)\right\|}_{2}^{2}\right].$$

The data x is perturbed with a noise distribution $p\left(\tilde{x}|x\right)$, which can be accurately modeled by the Gaussian mixture,

(14) $$p\left(\tilde{x}|x\right)=\int p\left(\sigma \right)G\;\left(\tilde{x};x,\sigma^{2}I\right)d\sigma ,$$

where $G\left(\tilde{x};x,\sigma^{2}I\right)$ is the Gaussian distribution with the variance $\sigma^{2}$. The Gaussian mixture enables parametric probability distributions to flexibly represent complex noise in real scenarios [15]. From Eq. (14), the gradient of logarithmic noise distribution can be calculated,

(15) $$\{\begin{array}{l} \nabla_{\tilde{x}}\text{log }p(\tilde{x}|x)=\int \lambda (\sigma ,\tilde{x}-x)\frac{x-\tilde{x}}{\sigma^{2}}d\sigma \\ \lambda (\sigma ,\tilde{x}-x)=\frac{p(\sigma )G\left(\tilde{x};x,\sigma^{2}I\right)}{\int p(\sigma )G\left(\tilde{x};x,\sigma^{2}I\right)d\sigma } \end{array}$$

From Eqs. (13) and (15), the score matching objective function in Eq. (13) is simplified to the following objective function:

(16) $$E_{p\left(\sigma \right)}E_{p\left(\tilde{x}|x\right)p_{\text{data}}\left(x\right)}\left(\lambda \left(\sigma ,\tilde{x}-x\right){\left\|S_{\theta }\left(\tilde{x}\right)+\frac{\tilde{x}-x}{\sigma^{2}}\right\|}_{2}^{2}\right)$$

Using machine learning techniques, the neural network $s_{\theta }\left(\tilde{x}\right)$ can be trained to establish score function model.

## 3. Experimental design and results

### 3.1. Dataset:

To evaluate the performance of the proposed ISM-based reconstruction method, we used the NIH-AAPM-Mayo Clinic CT Grand Challenge data as training dataset to establish the score function model. The dataset has 2,378 CT images with a slice thickness of 3mm from 10 patients and was randomly divided into a training dataset and a test dataset. The training dataset contains 1,923 images from 8 patients, and the test dataset has 455 images from the remaining 2 patients. The original image size is 512 × 512. The distance from the X-ray source focal spot to the isocenter of the imaging field of view is 54.1cm. The X-ray imaging geometry was set to a distance of 94.9 cm from the detector to the source. 888 detector elements with 0.1024cm pitch were equiangular distributed on a projection view. The X-ray CT imaging was simulated using the distance-driven algorithm. A total of 90 projection views are uniformly distributed over a 360-degree angular range to generate a few-view projection datasets. The projection datasets were corrupted by Poisson noise to simulate real x-ray imaging experiments for the tomographic image reconstruction.

### 3.2. Network training:

The standard training procedure was followed to perform the training, validation, and testing. We used the ResNet neural network to simulate the score function described in Section 2.3. The ResNet network are one residual block including three convolution layers with 64 filters of 7 × 7 kernels, followed by one residual block including three convolutional layers with 64 filters of 5 × 5 kernels, and one residual block including three convolution layers with 64 filters of 3 × 3 kernels. Each residual block works in a feed-forward fashion with the shortcut connection skipping three layers to implement an identity mapping. Then, one convolution layer with 64 filters of 3 × 3 kernels is performed, followed by one convolution layer with 32 filters of 3 × 3 kernels, and the last layer generates one feature map with a single 3 × 3 filter as the output. Every layer is followed by a ReLU unit. The ResNet network is trained using image patches of 64 × 64. Training the network is to find kernels in convolution layers to minimize loss function on a training dataset. The training procedure was programmed in Pytorch on a PC computer with an NVIDIA Titan XP GPU of 12 GB memory. The network parameters in the convolution kernels were randomly initialized according to the Gaussian distribution with mean of zero and variance of 0.01. The optimization was conducted using the ADAM algorithm with $\beta_{1}=0.9$, $\beta_{2}=0.999$. The network was trained at the learning rate of 10^−4^ using 1000 epochs. The training process took about 24 hours. As a result, the training process showed excellent convergence and stability. The trained neural network established the score function model for the image reconstruction in the iterative formula (8).

### 3.3. Evaluation by comparing with leading compressive sensing-based reconstruction methods:

**Fig. 1.**
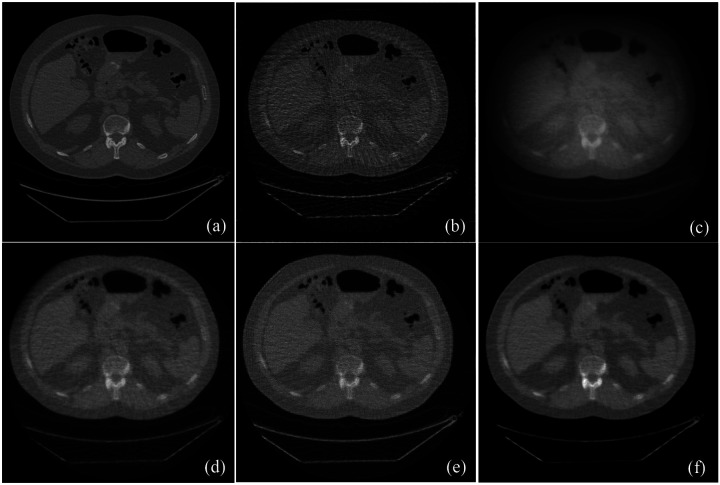
The comparison of images reconstruction between the ISM-based reconstruction method and leading reconstruction methods. (a) The ground truth image, (b) the image reconstructed from 90 projections using FBP, (c) the image reconstructed from 90 projections using the SART iteration, (d) the image reconstructed from 90 projections using the SART+TV, (e) the image reconstructed from 90 projections using the SART+LDMM, and (f) the image reconstructed from 90 projections using the ISM-based reconstruction method.

Based on the established score function model, we performed the image reconstruction from the test dataset of 90 projection views using the ISM-based reconstruction method. To compare the image quality with leading reconstruction methods, the image reconstructions were also performed from same dataset of 90 projection views using the standard filtered backprojection (FBP), simultaneous algebraic reconstruction technique (SART), SART with total variation (SART+TV) regularization, SART with low-dimensional manifold model (SART+LDMM) regularization methods. Figure 1 presented the images reconstructed by these reconstruction algorithms. Clearly, the proposed ISM-based reconstruction method well preserved structural information, especially texture features, achieving excellent spatial and contrast resolution while significantly reducing image noise. Statistically, 455 CT slices of 2 patients were reconstructed and quantitatively evaluated based on peak signal-to-noise ratio (PSNR) and structural similarity index (SSIM). The proposed ISM-based reconstruction method achieved average PSNR of 18.14dB±1.87 and SSIM of 0.7017±0.04, which are higher than the other reconstruction methods, as shown in Table 1.

### 3.4. Evaluation by comparing with state-of-the-art reconstruction methods on clinical data:

**Fig. 2.**
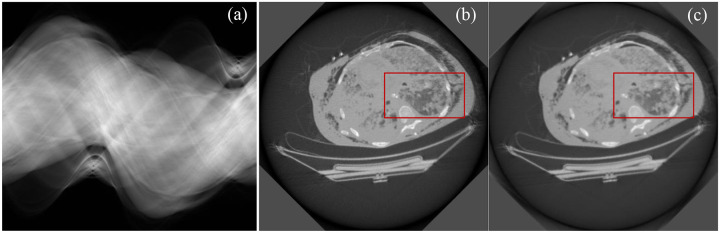

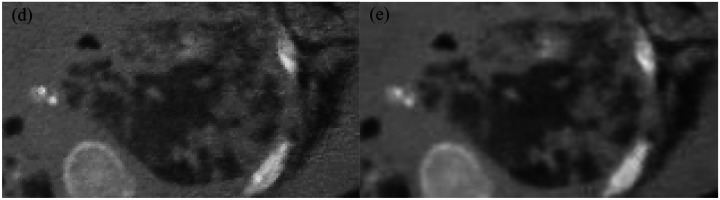
Image reconstruction from the sinogram of 984 views recorded from a clinical CT scanner. (a) The sinogram of 984 views, (b) the image reconstructed using FBP, (c) the image reconstructed using the ISM-based method, and (d-e) enlarged images for red rectangle in images (b-c), respectively.

The ISM-based image reconstruction method is evaluated by comparison with the filtered backprojection (FBP) analytical reconstruction algorithm, the gold standard for image reconstruction, and the score function-based image reconstruction methods [16]. A clinical raw dataset acquired from GE CT scanner was used to evaluate the ISM-based image reconstruction method. By processing for the projection data, we obtained a set of fan-beam sinogram, as shown in Fig. 2(a). CT X-ray imaging has a scanning trajectory with a radius of 54.1 cm. Source-to-detector distance is 94.9 cm. 984 projections are uniformly acquired over a 360-degree angular range. 888 detector elements with 0.1024 cm pitch were equiangular distributed on a projection view. The image matrix was of 512×512 pixels. From full view sinograms, the image reconstruction was performed using FBP and the ISM-based reconstruction method with the trained score function model, respectively. The results showed that the ISM-based image reconstruction method exhibits lower noise, as shown in Fig. 2(b–c).

**Fig. 3.**
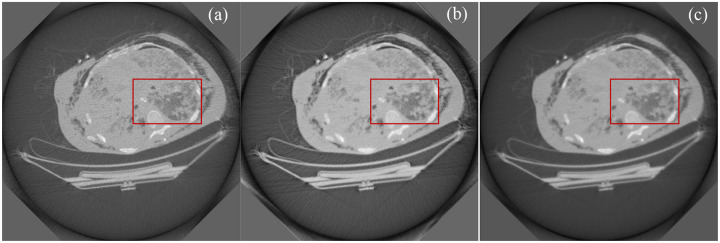

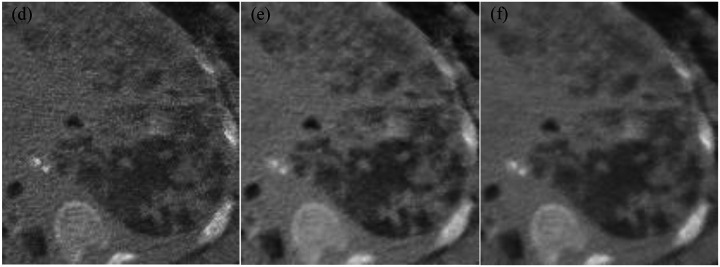
Comparison of CT image reconstructions from clinical raw data of 492 views. (a) The image reconstructed using SART, (b) the image reconstructed by the score function-based method [16], and (c) the image reconstructed by the ISM-based method, and (d-f) enlarged image for red rectangle in images (a-c), respectively.

In addition, we also performed image reconstruction from sinograms of 492 views using the ISM-based reconstruction method, scoring function-based reconstruction method [16], and SART method to compare the performance of these reconstruction methods. Results showed that the ISM-based image reconstruction presented lower noise and well preserved structural information, especially texture features, as shown in Fig. 3.

## 4. Discussions and conclusion

In this study, we model the X-ray projection data as the Poisson distribution, enabling us to reconstruct image using maximum a posteriori (MAP) estimation. The MAP estimation is to minimizing the image fidelity term while maximizing the logarithmic probability density of the image. The image reconstruction process relies on gradient-based optimization methods, which operate through an iterative procedure. The score function plays an important role in the image reconstruction by providing the gradient of the logarithmic probability density function. It incorporates statistical knowledge and serves as a key element in this iterative process, contributing to refine the reconstructed image for the each iteration. We have developed a new score matching solution for the image reconstruction by leveraging Gaussian mixture to characterize noise distributions. The improved score matching (ISM)-based image reconstruction presented lower noise and well preserved structural information, especially texture features. This ISM-based reconstruction method produces higher quality CT images comparing with popular score function-based method.

To accurately estimate the score function, we adopt a deep convolutional neural network (CNN) to simulate the score function, which is learned from the image dataset using a score matching method. The score matching-based image reconstruction method, facilitated by the learned score function, offers notable advantages for CT image reconstruction. In comparison to well-known techniques such as the simultaneous algebraic reconstruction technique (SART), as well as advanced methods like SART with total variation (TV) regularization, SART with low-dimensional manifold model (LDMM) regularization, and the score function-based method, our approach demonstrates improved image quality, which indicates that our score matching-based method surpasses these existing methods in terms of image quality metrics in low-dose or sparse-view CT scenarios.

Compressed sensing is based on the assumption that images are sparse or compressible in some transform domain, such as total variation, wavelet or Fourier transform. However, for some images, especially complex and finely structured biomedical images, this assumption cannot be accurately realized. In contrast, the score function approach provides a more flexible and data-driven prior information for image reconstruction, as it is learned directly from the data distribution. This allows for a more accurate representation of the underlying structure of the images.

In conclusion, we have formulated the reconstruction of CT images via an improved score-matching solution and demonstrated the feasibility and merits of this new method. The proposed score matching-based iterative method of image reconstruction has been theoretically demonstrated to converge to a minimum of the MAP objective. Moreover, the quantitative evaluation using PSNR and SSIM has shown that this method can achieve improved image quality in low dose or sparse-view CT. The proposed approach can reduce radiation dose and improve contrast resolution for the detection and characterization of lesions and other diseases. Further system evaluation and optimization studies are ongoing to explore the potential application of this new approach in CT imaging

## Figures and Tables

**Table 1.** Reconstructed image quality metrics

	Score function	FBP	SART	SART+TV	SART+LDMM
SSIM	0.7017	0.3703	0.5406	0.6724	0.5608
PSNR	18.1434	11.8587	16.797	17.1514	17.5293
